# Effect of Esketamine Nasal Spray on Cognition in Patients With Treatment-Resistant Depression: Results From Four Phase 3 Studies

**DOI:** 10.1093/ijnp/pyae046

**Published:** 2024-11-08

**Authors:** Randall L Morrison, Jaskaran Singh, Ella Daly, Maggie Fedgchin, Rachel Ochs-Ross, Keith Karcher, Rosanne Lane, Kim Cooper, Gerard Sanacora, Paul Maruff, Wayne C Drevets

**Affiliations:** Janssen Research & Development, LLC, Titusville, New Jersey, USA; Janssen Research & Development, LLC, San Diego, California, USA; Janssen Research & Development, LLC, Titusville, New Jersey, USA; Janssen Research & Development, LLC, Titusville, New Jersey, USA; Janssen Research & Development, LLC, Pennington, New Jersey, USA; Janssen Research & Development, LLC, Titusville, New Jersey, USA; Janssen Research & Development, LLC, Pennington, New Jersey, USA; Janssen Research & Development, LLC, Spring House, Pennsylvania, USA; Yale University, New Haven, Connecticut, USA; Cogstate Ltd, Melbourne, Australia; Janssen Research & Development, LLC, San Diego, California, USA

**Keywords:** Cognition, Cogstate battery test, esketamine, randomized controlled trials, treatment-resistant depression

## Abstract

**Background:**

While esketamine is effective in treatment-resistant depression (TRD), detailed information about the effect of esketamine on cognition is relatively scarce. This analysis assessed the effect of short-term (3 double-blind [DB] studies: DB1, DB2, and DB4) or long-term maintenance treatment (DB3) with esketamine nasal spray (ESK) compared with a placebo (PBO) combined with active-comparator, on cognition in patients with TRD.

**Methods:**

Patients (DB1/DB2/DB3: [18–64 years, n = 747]; DB4: [65 years or older, n = 137]) with TRD received ESK (DB1/DB2/DB3: 56/84 mg; DB4: 28/56/84 mg) or PBO+newly initiated oral antidepressant (OAD) as per treatment schedules. Cognitive assessments—Cogstate battery and Hopkins Verbal Learning Test-Revised—were administered at baseline, Day 28/early withdrawal, and follow-up visits in DB1/DB2/DB4 and at 12-week intervals in the DB3 maintenance phase. Descriptive statistics were used to analyze ESK effects on cognition with effect sizes and 95% confidence intervals to express the nature and magnitude of treatment effects relative to active-comparator+PBO. Correlation between depression severity (Montgomery-Ǻsberg Depression Rating Scale scores [MADRS]) and cognition was assessed at baseline and endpoint(s).

**Results:**

At baseline, mild-to-moderate impairment in psychomotor function, attention, and memory (working and episodic) were evident. For each DB1/DB2/DB4, group mean performance in Z-scores for ESK+OAD and OAD+PBO groups on all cognitive tests remained similar or slightly improved from baseline at endpoint (Day 28) and follow-up assessments. Similarly, in DB3 (maintenance phase), both groups generally showed improvement in cognitive performance at endpoint(s). Correlations between MADRS scores and performance on the cognitive test battery were small at baseline and endpoint(s).

**Conclusions:**

This analysis did not identify evidence of negative effects on cognition following short-term or long-term maintenance treatment with ESK+OAD in patients with TRD.

**Significance Statement**

Recently approved (by health authorities), esketamine nasal spray (ESK) demonstrated rapid and robust reduction of depressive symptoms, with a well-characterized manageable tolerability. This evidence for the robust efficacy of treatment with ESK raises the importance of considering other effects on the central nervous system, such as impact on cognition, associated with short-term or long-term treatment with ESK. To evaluate the effect of short-term and maintenance treatment with ESK on cognition in individuals with treatment-resistant depression (TRD), data were analyzed from 4 active-comparator-controlled phase 3 studies. The results did not indicate any negative effect on cognition. Additionally, the results showed improved function on several domains of cognition in this cohort of patients with TRD. Thus, the results of this analysis support that ESK for TRD, administered according to the short-term and long-term maintenance treatment study protocols, did not lead to negative cognitive effects.

## INTRODUCTION

The approval of the intranasal formulation of esketamine (esketamine nasal spray [ESK]; Spravato, Janssen Pharmaceuticals Inc., Titusville, NJ, USA) for therapeutic use in treatment-resistant depression (TRD) contributes substantially to emerging models of the role of N-methyl-D-aspartate (NMDA) neurotransmission in the pathophysiology of depression. In clinical trials, ESK produced a rapid and robust reduction in depressive symptoms in patients with TRD after 4 weeks of twice weekly treatment. Furthermore, these antidepressant effects could be maintained by repeated dosing administered at weekly or bi-weekly intervals (Daly et al., 2018, 2019). ESK combined with a newly initiated oral antidepressant (OAD) medicine has been approved for TRD by the health authorities in the United States (US FDA 2019), Europe (EMA 2019), and over 70 other countries.

The evidence of antidepressant efficacy with ESK increases the importance of considering other central nervous system effects, such as effect on cognition, with both short-term and long-term treatment (Sanacora et al., 2017; McIntyre et al., 2021). In studies with healthy volunteers, ketamine, a racemic mixture of R-ketamine and esketamine, repeatedly has been shown to induce acute and transient performance deficits on measures of visual and verbal memory (e.g., Zhornitsky et al., 2022). Similarly, among healthy volunteers, a single 84-mg dose of ESK resulted in a decline in performance across a range of cognitive domains (reaction time, visual recall, maze learning) at 40 minutes post dose relative to placebo; however, performance returned to placebo-comparable levels within 2 hours post dose (Morrison et al., 2018). Among long-term recreational users, however, ketamine has been associated with persistent deficits in episodic and semantic memory, although these observations were made in uncontrolled studies which generally involved polydrug users (Cheng et al., 2018). Given both the potential for adverse cognitive effects associated with moderate- and long-term ketamine use as well as the prevalence and functional impact of cognitive impairment associated with major depressive disorder (MDD), exploration of the effects of intranasal esketamine treatment in larger cohorts, studied over longer time intervals, using validated assessments of cognition is necessary.

The present report analyzed aggregate data from 4 phase 3, double-blind, multicenter ESK studies—TRANSFORM-1 (NCT02417064) (Fedgchin et al., 2019), TRANSFORM-2 (NCT02418585) (Popova et al., 2019), TRANSFORM-3 (NCT02422186) (Ochs-Ross et al., 2020), and SUSTAIN-1 (NCT02493868) (Daly et al., 2019) (hereafter referred to as DB1, DB2, DB4, and DB3, respectively). Data from these 4 studies in conjunction with data from SUSTAIN-2 (Wajs et al., 2020) [NCT02497287; the open-label (OL) long-term safety study] supported regulatory approval for use of ESK in TRD. Data from SUSTAIN-2 was not included in the current analysis due to the absence of a control arm, which is considered essential to evalaute the effects on logitudinal cognitive performance; moreover the cognitve assessments from that study are published eleswhere (Wajs et al., 2020). Each of the 4 studies included the same extensive cognitive test battery and studied large sample sizes compared with existing clinical trials in TRD. Individually, no study observed cognitive decline related to treatment with ESK. Furthermore, each of these clinical trials included an arm where placebo nasal spray (PBO) or ESK was added to the prescribed OAD for the entire study period. Thus, any estimates of ESK-related cognitive change in the TRD sample take into account the changes and variability in cognition that had occured over the time in TRD. However, the very large sample arising from aggregation of data across studies provides an opportunity to detect and characterize any effects of smaller magnitude from short-term or long-term treatment with ESK on cognition.

## METHODS

The data used for this analysis were from 4 phase 3 ESK studies: DB1 (Fedgchin et al., 2019), DB2 (Popova et al., 2019), DB3 (Daly et al., 2019), and DB4 (Ochs-Ross et al., 2020). DB1, DB2, and DB3 were conducted in patients aged 18–64 years with TRD, whereas DB4 was conducted in patients aged 65 years or older with TRD. All protocols and amendments were reviewed by an independent ethics committee or institutional review board at each site. The studies were conducted in accordance with the Declaration of Helsinki, Good Clinical Practice, and applicable regulatory requirements. Written informed consent was obtained from each patient before participating in the study. In these studies, cognition was measured as a secondary safety endpoint.

### Study Designs and Patient Population

**Figure 1.**
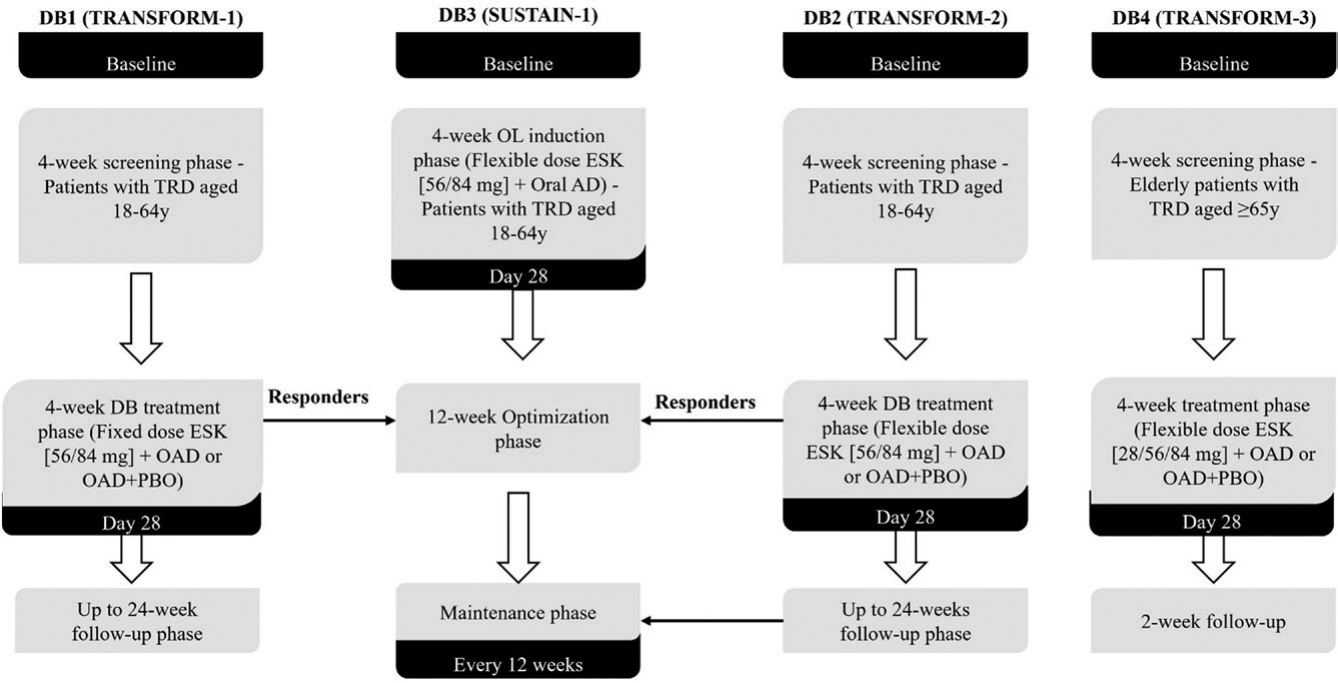
Study designs of the 4 ESK phase 3 studies. The timing of cognitive assessments is indicated by black boxes. In the DB1, DB2, and DB4 studies, cognitive assessments were performed at baseline, the endpoint of the double-blind treatment period (Day 28), the LOCF endpoint, Week 2 of the follow-up phase, and at the early withdrawal visit in the event of a patient’s early withdrawal from the study. In the DB3 study, cognitive assessments were administered at baseline and Day 28 for direct-entry patients; at 12-week intervals beginning from Week 32 through study endpoint or LOCF in the event of early withdrawal. At the end of the 4-week double-blind treatment phase, responders in DB1 and DB2 were eligible to enter DB3 (transferred-entry patients). DB3 had 5 phases including a 4-week screening phase, a 4-week OL induction phase (ESK 56 or 84 mg) + newly initiated OAD (for direct-entry patients only), a 12-week optimization phase to individualize treatment session frequency (once weekly or once every other week) for both direct-entry and transferred-entry patients, and a maintenance phase (for both direct-entry and transferred-entry patients) in which patients receiving ESK+OAD and in stable remission or stable response, were randomized to either continue ESK+OAD or be switched to OAD+PBO until relapse or study completion. DB, double-blind; ESK, esketamine nasal spray; LOCF, last observation carried forward; OAD, oral antidepressant; OL, open-label; PBO, placebo nasal spray; TRD, treatment-resistant depression.

Study designs and patient cohorts were previously published (Daly et al., 2019; Fedgchin et al., 2019; Popova et al., 2019; Ochs-Ross et al., 2020) and summarized in Figure 1, including timing of cognitive assessments. In total, cognitive data of 884 patients with TRD from these 4 studies were used for the analysis. The number of patients in DB1, DB2, DB3, and DB4 were 342 (ESK+OAD: 229; OAD+PBO: 113), 223 (ESK+OAD: 114; OAD+PBO: 109), 297 (ESK+OAD: 152 [of which 67 patients were transferred-entry from DB1/DB2]; OAD+PBO: 145 [of which 48 patients were transferred-entry from DB1/DB2]), and 137 (ESK+OAD: 72; OAD+PBO: 65), respectively (Table 1).

**Table 1.** Baseline demographics and clinical characteristics.

	DB1			DB2		DB4		DB3 (Maintenance phase)	
	ESK (56 mg)+OAD	ESK (84 mg)+OAD	OAD+PBO	ESK+OAD	OAD+PBO	ESK+OAD	OAD+ PBO	ESK+OAD	OAD+PBO
N	115	114	113	114	109	72	65	152	145
Age (y), mean (SD)	46.4 (11.18)	45.7 (11.10)	46.8 (11.36)	44.9 (12.58)	46.4 (11.14)	70.6 (4.79)	69.4 (4.15)	46.1 (11.67)	46.4 (10.58)
Sex, n (%)									
Male	34 (29.6)	35 (30.7)	32 (28.3)	39 (34.2)	46 (42.2)	27 (37.5)	25 (38.5)	56 (36.8)	44 (30.3)
Female	81 (70.4)	79 (69.3)	81 (71.7)	75 (65.8)	63 (57.8)	45 (62.5)	40 (61.5)	96 (63.2)	101 (69.7)
Race, n (%)									
Asian	2 (1.7)	1 (0.9)	2 (1.8)	1 (0.9)	1 (0.9)	0	0	0	1 (0.7)
Black or African American	7 (6.1)	7 (6.1)	5 (4.4)	6 (5.3)	5 (4.6)	0	0	6 (3.9)	7 (4.8)
American Indian or Alaskan Native	0	0	0	0	0	0	0	0	1 (0.7)
White	91 (79.1)	85 (74.6)	86 (76.1)	106 (93.0)	102 (93.6)	66 (91.7)	64 (98.5)	137 (90.1)	131 (90.3)
Other	8 (7.0)	12 (10.5)	10 (8.8)	0	0	0	0	5 (3.3)	2 (1.4)
Multiple	0	0	1 (0.9)	1 (0.9)	1 (0.9)	4 (5.6)	0	1 (0.7)	1 (0.7)
Not reported	7 (6.1)	9 (7.9)	9 (8.0)	0	0	1 (1.4)	1 (1.5)	3 (2.0)	2 (1.4)
Unknown	0	0	0	0	0	1 (1.4)	0	0	0
Class of OAD, n (%)									
SNRI	65 (56.5)	67 (58.8)	64 (56.6)	77 (67.5)	75 (68.8)	31 (43.1)	30 (46.2)	97 (63.8)	94 (64.8)
SSRI	50 (43.5)	47 (41.2)	49 (43.4)	37 (32.5)	34 (31.2)	41 (56.9)	35 (53.8)	55 (36.2)	51 (35.2)
OAD, n (%)									
Duloxetine	49 (42.6)	43 (37.7)	44 (38.9)	60 (52.6)	61 (56.0)	25 (34.7)	23 (35.4)	74 (48.7)	68 (46.9)
Escitalopram	26 (22.6)	23 (20.2)	24 (21.2)	21 (18.4)	17 (15.6)	25 (34.7)	25 (38.5)	30 (19.7)	24 (16.6)
Sertraline	24 (20.9)	24 (21.1)	25 (22.1)	16 (14.0)	16 (14.7)	15 (20.8)	10 (15.4)	25 (16.4)	27 (18.6)
Venlafaxine XR	16 (13.9)	24 (21.1)	20 (17.7)	17 (14.9)	15 (13.8)	7 (9.7)	7 (10.8)	23 (15.1)	26 (17.9)
Age when diagnosed with MDD (y), mean (SD)	30.3 (12.34)	32.1 (12.86)	31.8 (12.44)	32.1 (12.53)	35.3 (13.04)	42.6 (16.18)	43.7 (16.28)	34.0 (12.29)	33.6 (11.03)
Baseline MADRS total score, mean (SD)	37.4 (4.76)	37.8 (5.58)	37.5 (6.16)	37.0 (5.69)	37.3 (5.66)	35.5 (5.91)	34.8 (6.44)	38.5 (5.50)	38.2 (4.79)
Duration of current episode (weeks), mean (SD)	202.8 (277.25)	212.7 (327.62)	193.1 (264.10)	111.4 (124.28)	118.0 (187.37)	163.1 (277.04)	274.1 (395.47)	116.0 (180.27)	123.2 (197.83)

Abbreviations: ESK, esketamine nasal spray; MADRS, Montgomery-Ǻsberg Depression Rating Scale; MDD, major depressive disorder; OAD, oral antidepressant; PBO, placebo nasal spray; SD, standard deviation; SNRI, serotonin and norepinephrine reuptake inhibitor; SSRI, selective serotonin reuptake inhibitor; XR, extended-release; y, year.

Studies DB1, DB2, and DB4 each comprised 3 phases: (1) screening/prospective observation phase (4 weeks); (2) double-blind treatment phase (4 weeks); and (3) post-treatment follow-up phase (DB1/DB2: up to 24 weeks; DB4: 2 weeks). Patients who completed the 4-week treatment phase were classified as responders if they had ≥50% reduction from baseline in the Montgomery-Ǻsberg Depression Rating Scale (MADRS) total score (Montgomery and Asberg, 1979). Responders from the DB1 and DB2 studies were eligible to enter the long-term DB3 study (i.e., “transferred-entry” patients).

The DB3 study comprised up to 5 phases: (1) screening/prospective observation phase (4 weeks; direct-entry patients only); (2) OL induction phase (4 weeks; direct-entry patients only); (3) optimization phase (12 weeks; OL for direct-entry patients; double-blind for DB1 and DB2 transferred-entry patients); (4) double-blind maintenance phase (varying duration; randomized withdrawal or event driven); and (5) follow-up phase (2 weeks post-treatment). For DB3, only data from patients entering the double-blind maintenance phase are included in the current analysis.

### Treatments

In DB1, DB2, and DB4, ESK or PBO plus a newly initiated daily OAD was administered twice weekly for 4 weeks with a fixed ESK dose (DB1: 56 or 84 mg) or flexible ESK dose (DB2 and DB4: 28 [DB4 only], 56/84 mg). In DB3, direct-entry patients received a flexible ESK dose (56/84 mg) twice weekly plus a newly initiated OAD during the 4-week OL induction phase. In the 12-week optimization phase of DB3, ESK dosing frequency was reduced to once weekly for 4 weeks then individualized to once weekly or every 2 weeks based on severity of depressive symptoms. At the start of the maintenance phase of DB3, patients receiving ESK+OAD who achieved stable remission or stable response at the end of the optimization phase were randomized 1:1 to either continue ESK+OAD or switch to OAD+PBO. Per study design, DB3 continued until the number of relapses intended for efficacy analyses had occurred during the maintenance phase (i.e., the study was stopped based on predetermined stopping rules based on sample size estimation).

For the newly initiated OAD in all 4 studies, before initiating nasal spray treatment, investigators selected 1 from among 4 ADs (that were not previously) used in the current depressive episode: escitalopram, duloxetine, sertraline, or venlafaxine extended-release; OAD was administered daily in an OL manner and was to be continued throughout the study.

### Cognitive Assessment

Cognition was assessed in each study using the same neuropsychological tests. The cognitive domains assessed were chosen based on the following criteria: (1) shown to be impaired in patients with depression (Davis et al., 2017; Chen et al., 2018); or (2) shown to be influenced by the treatment with intravenous ketamine in TRD (Murrough et al., 2015); or (3) demonstrated sensitivity to the acute effect of a single 84-mg dose of ESK in healthy adults (Morrison et al., 2018).

Seven domains of cognition were measured using 5 tests from the Cogstate computerized test battery (hereafter termed the Cogstate battery) (Fredrickson et al., 2010) and 2 scores from the Hopkins Verbal Learning Test-Revised (HVLT-R) (Benedict et al., 1998). Each of these cognitive tests has been described in detail previously (Morrison et al., 2018). Briefly, the Cogstate battery included the following tests: Detection (DET) to measure simple reaction time (psychomotor function), Identification (IDN) to measure choice reaction time (attention), One-Card Learning (OCL) to measure visual learning, One Back Memory (ONB) to measure working memory, and Groton Maze Learning (GML) to measure executive function. The HVLT-R (including Total Recall and Delayed Recall [HVLT-R_D]) was used to measure the aspects of verbal episodic memory. For the main performance measure for each test, standardized scores (Z-scores) were derived by subtracting the mean of the age-stratified population normative data from the patient’s raw score and dividing this by the standard deviation (SD) of the same age-stratified normative data. For tests where lower scores indicated faster reaction times (DET, IDN, OCL) or fewer errors (GML), the sign (positive or negative) of the Z-score values and changes in the Z-scores were reversed so that for all cognitive tests positive values (including change from baseline values) indicated better performance. This adjustment also systematized scores reflecting differences in the change from baseline scores between treatment groups so that positive values indicated the change under ESK was greater or better than PBO and vice versa.

In the DB1, DB2, and DB4 studies, the mean Z-scores were assessed at predose baseline, the endpoint of the double-blind treatment period (Day 28), the last observation carried forward (LOCF) endpoint, Week 2 of the follow-up phase, and at the early withdrawal visit in the event of a patient’s early withdrawal from the study. Also, mean changes from baseline at Day 28 and LOCF endpoint were determined. In the DB3 study, cognitive assessments were administered at baseline and Day 28 for direct-entry patients, and at 12-week intervals beginning from Week 32 of the maintenance phase through the study endpoint or last study visit in the event of early withdrawal. DB3 was a relapse prevention study in which patients were considered to have completed the maintenance phase upon relapse, discontinuation of treatment, or when the study stopped after the specified number of relapse events was reached, leading to different durations for patients in the maintenance phase. As such, data from patients’ cognitive assessments considered in the current analyses were obtained from the assessments completed by the patient at “end of maintenance phase” visit. Additional analyses of results at the Week 32, 44, and 56 visits were also performed.

### Data Analytic Strategy

In the 4 ESK phase 3 studies, cognitive tests were included as secondary safety endpoints and hence were not the focus of computations of necessary statistical power. Consequently, in the submissions to regulatory authorities and in the related reports, inferences regarding the cognitive effects of ESK were based on descriptive statistics only (Daly et al., 2019; Fedgchin et al., 2019; Popova et al., 2019; Ochs-Ross et al., 2020). In the current analyses, aggregation of data across studies allowed extension of this original analytic strategy. All analyses were conducted on the safety analysis set, which included all randomized patients who received at least 1 dose of ESK or OAD during the double-blind treatment phase (DB1/DB2/DB4) or the maintenance phase (DB3). As the entry criteria for the DB1 and DB2 studies were the same, study data were combined (DB1+DB2 pooled) for each cognitive test. In addition, a series of data analytic strategies were followed. First, group means and SDs of Z-scores and change from baseline Z-scores were calculated for each cognitive test for treatment groups for each study assessment visit. Second, measures of effect size, and their associated 95% confidence intervals (CIs), were used to guide conclusions about the effect of ESK on each cognitive test. Analysis of covariance (ANCOVA) was used to estimate the magnitude of treatment group differences for each cognitive test. For the DB1+DB2 pooled data and the DB4 data, the factors in the ANCOVA model included treatment group, region, class of coadministered OAD (serotonin and norepinephrine reuptake inhibitor or selective serotonin reuptake inhibitor), and study (DB1+DB2 pooled only), with a baseline value of the cognitive outcome as a covariate. For the DB3 data, the factors in the ANCOVA model included treatment group and country, with baseline value of the cognitive outcome as a covariate. If the 95% CIs associated with a treatment group difference did not include zero in DB3, the treatment groups were classified as being different on the cognitive test at the end of maintenance phase visit. Group means and SDs of Z-scores and change from baseline Z-scores were also calculated for each cognitive test at the Week 32, 44, and 56 visits. Given the variable duration of the maintenance phase for each patient, a mixed effects model for repeated measures (MMRM) analysis was then conducted to explore the length of time in study for “end of maintenance” visits. To analyze this time-in-study data, the relative day in the maintenance phase of a cognitive assessment was assigned to a visit interval (supplementary Table 1), with, for example, assessments between Day 92 and Day 323 classified as occurring at Week 32, 44, or 56 visits. Because sample sizes were substantially reduced for these later visits, especially in the OAD+PBO group, statistical significance was not computed for treatment group differences at these visits. Instead, the magnitude of group differences between the modeled means from the MMRM, and their associated 95% CIs, at these later visits were used to guide interpretation.

In addition to analyses of change in cognition over time, baseline cognitive test data from the phase 3 studies provided an opportunity to determine the magnitude of cognitive impairment in TRD, and the extent to which it was associated with depression severity, before any study-related treatment. To evaluate relationships between depressive symptom severity and cognition, Pearson correlation coefficients were calculated between the MADRS total score and each standardized cognitive test performance measure from the baseline assessment, change from baseline at Day 28, and change from baseline at the endpoint in the 3 short-term studies (DB1, DB2, and DB4).

## RESULTS

Demographic and baseline characteristics for each study (DB1, DB2, DB3, and DB4) were previously described (Daly et al., 2019; Fedgchin et al., 2019; Popova et al., 2019; Ochs-Ross et al., 2020) and are summarized in Table 1. Overall, the demographic and baseline clinical characteristics were balanced between the ESK+OAD and OAD+PBO groups within each study.

**Figure 2.**
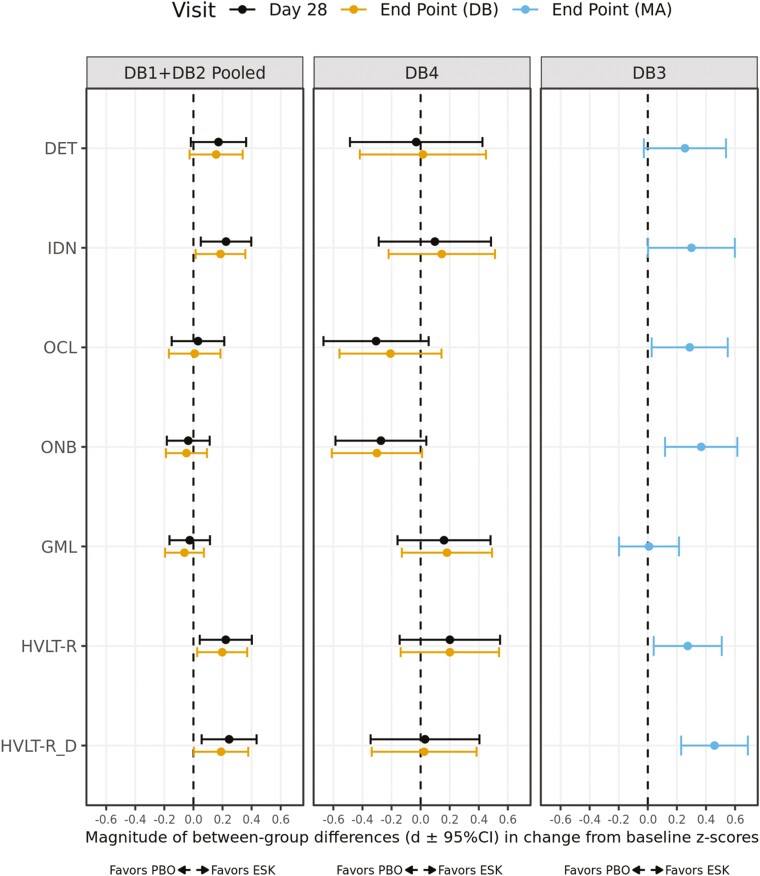
Magnitude of between-group treatment effects (d ± 95% CI) in mean change from baseline Z-scores for DB1+DB2 pooled, DB4, and DB3 (safety analysis set). DB1+DB2 pooled and DB4: Between-treatment differences and 95% CIs were estimated from ANCOVA models with factors for study, treatment group, region, class of AD, and baseline value of the cognitive test as covariate. DB3: Between-treatment differences and 95% CIs were estimated from an ANCOVA model with factors for treatment group, country, and baseline value of the cognitive test as covariate. Endpoint (DB): LOCF endpoint of the double-blind phase (DB1, DB2, DB4); Endpoint (MA): LOCF endpoint of the maintenance phase (DB3). AD, antidepressant; ANCOVA, analysis of covariance; CI, confidence interval; DET, Detection; ESK, esketamine nasal spray; GML, Groton Maze Learning; HVLT-R, Hopkins Verbal Learning Test-Revised; HVLT-R_D, HVLT-R Delayed Recall; IDN, Identification; LOCF, last observation carried forward; OCL, One-Card Learning; ONB, One Back Memory; PBO, placebo nasal spray.

Table 2 summarizes the descriptive statistics of cognitive test Z-scores for the DB1+DB2 pooled data. Figure 2 provides a summary of the magnitude and direction of effect sizes for differences between ESK+OAD and OAD+PBO groups for each cognitive test for the DB1+DB2 pooled and DB4 and DB3 studies. Performance on all cognitive tests, except the HVLT-R for the OAD+PBO group, improved from baseline for both ESK+OAD and OAD+PBO groups at Day 28 or study endpoint (Table 2). For the HVLT-R tests, performance in the OAD+PBO group declined slightly (<0.1 SD units) at Day 28 and at the study endpoint. The model-derived between-treatment differences in change from baseline Z-scores varied in their signs and were small in magnitude (~ ±0.2). For the IDN, HVLT-R, and HVLT-R_D tests, the 95% CIs for the small but positive effect sizes did not include zero, and therefore performance in the ESK+OAD group was classified as different (i.e., toward greater improvement) than the OAD+PBO group.

**Table 2.** Descriptive statistics of cognitive test Z-scores for pooled DB1+DB2, DB4, and DB3 (maintenance phase) studies (safety analysis set).

Test	Timepoint	Treatment group	N	Mean Z-score (SD)			Between-treatment difference, d (95% CI) in mean change from baseline Z-score
				Baseline assessment	Follow-up visit assessment	Change from baseline	
DB1+DB2 Pooled							
DET	Day 28	ESK+OAD	278	-0.887 (1.602)	-0.493 (1.313)	0.394 (1.317)	0.172 (-0.018, 0.362)
		OAD+PBO	192	-0.887 (1.633)	-0.643 (1.382)	0.244 (1.247)	
	Endpoint	ESK+OAD	304	-0.848 (1.600)	-0.484 (1.327)	0.364 (1.284)	0.156 (-0.027, 0.338)
		OAD+PBO	201	-0.843 (1.627)	-0.620 (1.393)	0.223 (1.237)	
IDN	Day 28	ESK+OAD	286	-0.559 (1.500)	-0.269 (1.259)	0.290 (1.121)	0.224 (0.051, 0.398)
		OAD+PBO	194	-0.756 (1.773)	-0.575 (1.469)	0.182 (1.188)	
	Endpoint	ESK+OAD	312	-0.526 (1.484)	-0.279 (1.270)	0.248 (1.139)	0.186 (0.015, 0.357)
		OAD+PBO	203	-0.698 (1.774)	-0.535 (1.476)	0.163 (1.190)	
OCL	Day 28	ESK+OAD	293	-0.456 (1.166)	-0.182 (1.223)	0.274 (1.117)	0.031 (-0.149, 0.212)
		OAD+PBO	197	-0.512 (1.126)	-0.227 (1.112)	0.285 (1.120)	
	Endpoint	ESK+OAD	319	-0.434 (1.155)	-0.204 (1.214)	0.230 (1.120)	0.009 (-0.168, 0.186)
		OAD+PBO	207	-0.512 (1.129)	-0.244 (1.138)	0.268 (1.140)	
ONB	Day 28	ESK+OAD	291	-0.743 (1.324)	-0.514 (1.142)	0.229 (0.953)	-0.036 (-0.183, 0.112)
		OAD+PBO	196	-0.881 (1.533)	-0.536 (1.299)	0.345 (0.976)	
	Endpoint	ESK+OAD	317	-0.710 (1.313)	-0.493 (1.130)	0.217 (0.941)	-0.048 (-0.189, 0.093)
		OAD+PBO	206	-0.832 (1.521)	-0.496 (1.293)	0.335 (0.964)	
GML	Day 28	ESK+OAD	277	-0.089 (0.913)	0.024 (0.971)	0.113 (0.819)	-0.025 (-0.165, 0.114)
		OAD+PBO	186	-0.118 (0.942)	0.062 (0.956)	0.180 (0.773)	
	Endpoint	ESK+OAD	302	-0.072 (0.905)	0.020 (0.959)	0.092 (0.807)	-0.061 (-0.195, 0.072)
		OAD+PBO	195	-0.126 (0.952)	0.067 (0.945)	0.193 (0.770)	
HVLT-R Total Recall	Day 28	ESK+OAD	303	-0.612 (1.271)	-0.532 (1.419)	0.081 (1.100)	0.222 (0.043, 0.401)
		OAD+PBO	204	-0.636 (1.246)	-0.713 (1.225)	-0.077 (1.028)	
	Endpoint	ESK+OAD	328	-0.595 (1.266)	-0.539 (1.397)	0.057 (1.103)	0.198 (0.026, 0.370)
		OAD+PBO	214	-0.631 (1.241)	-0.709 (1.213)	-0.078 (1.011)	
HVLT-R_D	Day 28	ESK+OAD	301	-0.616 (1.281)	-0.497 (1.364)	0.119 (1.151)	0.245 (0.056, 0.434)
		OAD+PBO	204	-0.639 (1.326)	-0.732 (1.367)	-0.093 (1.105)	
	Endpoint	ESK+OAD	326	-0.604 (1.286)	-0.535 (1.380)	0.069 (1.185)	0.190 (0.003, 0.377)
		OAD+PBO	214	-0.633 (1.323)	-0.723 (1.365)	-0.090 (1.108)	
DB4							
DET	Day 28	ESK+OAD	56	-0.544 (1.394)	-0.707 (1.635)	-0.163 (1.271)	-0.030 (-0.486, 0.425)
		OAD+PBO	58	-0.103 (1.272)	-0.327 (1.398)	-0.224 (1.221)	
	Endpoint	ESK+OAD	61	-0.493 (1.415)	-0.623 (1.630)	-0.131 (1.244)	0.015 (-0.418, 0.449)
		OAD+PBO	60	-0.074 (1.276)	-0.309 (1.388)	-0.235 (1.202)	
IDN	Day 28	ESK+OAD	56	-0.559 (1.398)	-0.490 (1.562)	0.069 (1.228)	0.098 (-0.288, 0.484)
		OAD+PBO	58	-0.107 (1.174)	-0.233 (1.170)	-0.126 (0.869)	
	Endpoint	ESK+OAD	61	-0.482 (1.384)	-0.393 (1.542)	0.089 (1.185)	0.146 (-0.220, 0.511)
		OAD+PBO	60	-0.057 (1.191)	-0.203 (1.184)	-0.146 (0.870)	
OCL	Day 28	ESK+OAD	56	-0.837 (1.144)	-0.921 (1.291)	-0.084 (1.048)	-0.306 (-0.668, 0.055)
		OAD+PBO	58	-0.559 (1.296)	-0.440 (1.273)	0.119 (0.969)	
	Endpoint	ESK+OAD	61	-0.823 (1.110)	-0.855 (1.270)	-0.031 (1.048)	-0.207 (-0.558, 0.143)
		OAD+PBO	60	-0.587 (1.311)	-0.481 (1.295)	0.106 (0.956)	
ONB	Day 28	ESK+OAD	58	-0.442 (1.435)	-0.417 (1.485)	0.026 (0.940)	-0.273 (-0.585, 0.039)
		OAD+PBO	56	-0.167 (1.316)	0.121 (1.193)	0.288 (0.829)	
	Endpoint	ESK+OAD	63	-0.431 (1.524)	-0.380 (1.531)	0.051 (0.923)	-0.300 (-0.611, 0.010)
		OAD+PBO	58	-0.150 (1.321)	0.169 (1.200)	0.320 (0.883)	
GML	Day 28	ESK+OAD	41	-0.065 (1.011)	0.214 (0.859)	0.279 (0.795)	0.160 (-0.159, 0.480)
		OAD+PBO	41	0.153 (0.872)	0.216 (1.035)	0.063 (0.777)	
	Endpoint	ESK+OAD	44	-0.046 (0.978)	0.242 (0.843)	0.288 (0.777)	0.181 (-0.129, 0.491)
		OAD+PBO	42	0.158 (0.862)	0.209 (1.024)	0.051 (0.771)	
HVLT-R Total Recall	Day 28	ESK+OAD	60	-0.513 (1.093)	-0.488 (1.063)	0.025 (0.877)	0.201 (-0.144, 0.546)
		OAD+PBO	59	-0.153 (1.180)	-0.417 (1.356)	-0.264 (1.096)	
	Endpoint	ESK+OAD	65	-0.425 (1.124)	-0.444 (1.110)	-0.019 (0.921)	0.201 (-0.137, 0.540)
		OAD+PBO	61	-0.167 (1.174)	-0.443 (1.365)	-0.277 (1.083)	
HVLT-R_D	Day 28	ESK+OAD	60	-0.558 (1.147)	-0.634 (1.178)	-0.076 (1.056)	0.030 (-0.344, 0.404)
		OAD+PBO	59	-0.322 (1.262)	-0.463 (1.476)	-0.141 (1.065)	
	Endpoint	ESK+OAD	65	-0.471 (1.153)	-0.590 (1.197)	-0.119 (1.051)	0.024 (-0.336, 0.384)
		OAD+PBO	61	-0.348 (1.290)	-0.502 (1.483)	-0.154 (1.055)	
DB3							
DET	Endpoint	ESK+OAD	137	-1.053 (1.808)	-0.618 (1.294)	0.435 (1.694)	0.255 (-0.027, 0.538)
		OAD+PBO	133	-1.080 (1.681)	-0.860 (1.517)	0.220 (1.349)	
IDN	Endpoint	ESK+OAD	139	-0.758 (1.783)	-0.489 (1.436)	0.269 (1.606)	0.300 (0.002, 0.598)
		OAD+PBO	135	-0.783 (1.768)	-0.800 (1.621)	-0.016 (1.396)	
OCL	Endpoint	ESK+OAD	141	-0.568 (1.316)	-0.053 (1.167)	0.516 (1.270)	0.288 (0.027, 0.550)
		OAD+PBO	138	-0.436 (1.160)	-0.278 (1.330)	0.157 (1.268)	
ONB	Endpoint	ESK+OAD	140	-0.728 (1.544)	-0.329 (1.190)	0.399 (1.367)	0.367 (0.118, 0.615)
		OAD+PBO	138	-0.852 (1.475)	-0.762 (1.432)	0.090 (1.079)	
GML	Endpoint	ESK+OAD	126	-0.061 (0.945)	0.253 (0.925)	0.315 (0.758)	0.008 (-0.198, 0.214)
		OAD+PBO	133	-0.407 (1.621)	0.102 (1.083)	0.509 (1.399)	
HVLT-R Total Recall	Endpoint	ESK+OAD	145	-0.858 (1.283)	-0.063 (1.246)	0.794(1.062)	0.274 (0.040, 0.508)
		OAD+PBO	140	-0.958 (1.338)	-0.386 (1.417)	0.572 (1.086)	
HVLT-R_D	Endpoint	ESK+OAD	145	-0.860 (1.272)	-0.119 (1.093)	0.741 (1.077)	0.459 (0.229, 0.688)
		OAD+PBO	140	-0.776 (1.477)	-0.521 (1.358)	0.255 (1.251)	

Abbreviations: ANCOVA, analysis of covariance; CI, confidence interval; DB, double-blind; DET, Detection; ESK, esketamine nasal spray; GML, Groton Maze Learning; HVLT-R, Hopkins Verbal Learning Test-Revised; HVLT-R_D, HVLT-R Delayed Recall; IDN, Identification; LOCF, last observation carried forward; OAD, oral antidepressant; OCL, One-Card Learning; ONB, One Back Memory; PBO, placebo nasal spray; SD, standard deviation; SNRI, serotonin and norepinephrine reuptake inhibitor; SSRI, selective serotonin reuptake inhibitor.

All group mean values are computed on age standardized scores. A negative group mean indicates performance is worse than age-stratified normative data and vice versa. A negative group mean change indicates performance has declined from baseline and vice versa. A negative difference value indicates change in performance under ESK+OAD was worse than change in performance under OAD+PBO and vice versa.

Between-treatment differences and 95% CIs were estimated from ANCOVA models including factors for treatment group, region, class of co-administered OAD (SNRI or SSRI), and study (DB1 or DB2), with baseline value of the cognitive test as a covariate. Day 28 assessment is the end of the 4-week double-blind phase and endpoint assessments are at LOCF in the double-blind phase. For DB3, endpoint assessment is the LOCF endpoint of the maintenance phase.

A qualitatively similar pattern of effects on cognition was observed in DB4 for patients aged 65 years or older (Table 2). However, for this age group, only performance on the ONB and GML tests improved from baseline for both ESK+OAD and OAD+PBO groups at Day 28 or the study endpoint. Treatment differences on these cognitive tests were small in magnitude (~ ±0.3), and their associated 95% CIs included zero. Hence, it was considered that there was no difference between ESK+OAD and OAD+PBO groups for these measures (Figure 2).

Table 2 also summarizes the descriptive statistics of the cognitive test Z-scores for younger (aged 18–64 years) patients who proceeded to the maintenance phase of the DB3. Performance on all cognitive tests (except the IDN test for the OAD+PBO group) improved from baseline to the study endpoint for both ESK+OAD and OAD+PBO groups. For the IDN test, performance in the OAD+PBO group had declined slightly (<0.1 SD units) at the study endpoint. Model-derived between-treatment differences and their 95% CIs indicated that the performance of ESK+OAD group was superior to the OAD+PBO group for the IDN, OCL, ONB, HVLT-R, and HVLT-R_D tests (i.e., 95% CIs did not include zero).

Descriptive statistics of cognitive test Z-scores at Week 32, 44, and 56 of the DB3 maintenance phase are shown in Table 3. Assessments at Week 32, 44, and 56 demonstrated that improvement from baseline on all cognitive tests for both ESK+OAD and OAD+PBO groups occurred across these 3 timepoints. In addition, improvement from baseline in the ESK+OAD group was numerically better than in the OAD+PBO group at the Week 32 and Week 44 assessment for each cognitive test, except the IDN at Week 32 and GML at Week 32 and Week 44, where the sample sizes retained were sufficient for accurate estimations of mean changes of Z-scores. Notably, at Week 56, improvement in the ESK+OAD group appeared less than in the OAD+PBO group; however, this finding should be interpreted with caution due to the small sample size at that timepoint. Despite the small sample sizes at the study visits analyzed, particularly in the OAD+PBO group, and for the Week 56 visit, the findings from the MMRM analyses of differences between the ESK+OAD and OAD+PBO groups at the Week 32, 44, and 56 visits were consistent with the outcomes from the ANCOVA analyses of DB3 (supplementary Table 2).

**Table 3.** Descriptive statistics of cognitive test Z-scores at Week 32, 44, and 56 for the DB3 study (maintenance phase only; safety analysis set).

Test	Treatment group	Week 32 Mean Z-score (SD)				Week 44 Mean Z-score (SD)				Week 56 Mean Z-score (SD)			
		N	Baseline assessment	Follow-up visit assessment	Change from baseline	N	Baseline assessment	Follow-up visit assessment	Change from baseline	N	Baseline assessment	Follow-up visit assessment	Change from baseline
DET	ESK+OAD	74	-1.299 (1.954)	-0.690 (1.510)	0.609 (1.549)	35	-1.596 (2.262)	-0.679 (1.270)	0.917 (2.044)	9	-0.891 (1.908)	-0.7342 (1.392)	0.157 (1.368)
	OAD+PBO	43	-1.328 (1.841)	-0.835 (1.187)	0.493 (1.471)	23	-1.248 (2.030)	-0.950 (1.649)	0.299 (1.145)	9	-2.178 (2.470)	-0.959 (1.932)	1.220 (1.627)
IDN	ESK+OAD	76	-1.031 (1.945)	-0.508 (1.507)	0.523 (1.468)	36	-1.318 (2.012)	-0.860 (1.485)	0.458 (1.522)	21	-0.645 (1.591)	-0.534 (1.188)	0.111 (1.075)
	OAD+PBO	44	-1.070 (1.965)	-0.517 (1.446)	0.553 (1.367)	23	-1.130 (2.126)	-0.755 (1.977)	0.375 (1.067)	9	-1.792 (2.332)	-1.214 (2.231)	0.578 (0.987)
OCL	ESK+OAD	78	-0.613 (1.358)	-0.079 (1.297)	0.534 (1.566)	37	-0.720 (1.105)	-0.356 (1.130)	0.363 (1.106)	22	-0.392 (1.070)	-0.349 (1.048)	0.042 (0.923)
	OAD+PBO	45	-0.621 (1.290)	-0.145 (1.287)	0.477 (1.237)	23	-0.318 (1.028)	-0.066 (1.209)	0.252 (1.018)	9	-0.195 (0.854)	0.194 (1.089)	0.389 (0.818)
ONB	ESK+OAD	78	-0.838 (1.594)	-0.406 (1.125)	0.433 (1.262)	36	-1.175 (1.716)	-0.546 (1.091)	0.629 (1.141)	22	-0.410 (1.201)	-0.177 (0.721)	0.233 (0.802)
	OAD+PBO	45	-0.858 (1.544)	-0.771 (1.391)	0.087 (1.087)	23	-1.075 (1.790)	-0.844 (1.769)	0.231 (1.002)	9	-1.195 (1.770)	-0.883 (2.152)	0.312 (1.492)
GML	ESK+OAD	74	-0.101 (1.010)	0.210 (1.182)	0.312 (1.216)	33	0.050 (0.928)	0.241 (0.895)	0.190 (0.802)	20	0.129 (0.685)	0.363 (0.843)	0.233 (0.879)
	OAD+PBO	41	-0.186 (0.759)	0.306 (0.678)	0.492 (0.641)	21	-0.274 (0.752)	0.348 (0.738)	0.622 (0.681)	8	-0.218 (0.628)	0.371 (0.628)	0.590 (0.713)
HVLT-R Total Recall	ESK+OAD	85	-0.977 (1.310)	-0.140 (1.204)	0.837 (1.024)	43	-0.916 (1.351)	-0.011 (1.265)	0.904 (1.211)	22	-0.689 (1.151)	0.009 (1.342)	0.698 (0.904)
	OAD+PBO	49	-1.172 (1.317)	-0.636 (1.330)	0.536 (1.102)	25	-1.223 (1.298)	-0.431 (1.616)	0.792 (1.182)	10	-1.789 (1.285)	-0.948 (1.504)	0.841 (1.010)
HVLT-R_D	ESK+OAD	85	-1.043 (1.280)	-0.241 (1.089)	0.802 (1.105)	43	-1.078 (1.430)	-0.154 (1.212)	0.924 (1.111)	22	-0.748 (1.272)	-0.076 (1.161)	0.671 (0.816)
	OAD+PBO	49	-1.067 (1.231)	-0.614 (1.201)	0.453 (1.045)	25	-1.220 (1.361)	-0.325 (1.385)	0.895 (0.915)	10	-1.776 (1.464)	-0.843 (1.456)	0.934 (1.054)

Abbreviations: DET, Detection; ESK, esketamine nasal spray; GML, Groton Maze Learning; HVLT-R, Hopkins Verbal Learning Test-Revised; HVLT-R_D, HVLT-R Delayed Recall; IDN, Identification; LOCF, last observation carried forward; OAD, oral antidepressant; OCL, One-Card Learning; ONB, One Back Memory; PBO, placebo nasal spray; SD, standard deviation.

All group mean values are computed on age standardized Z-scores. A negative group mean indicates performance is worse than age-stratified normative data and vice versa. A negative group mean change indicates performance has declined from baseline and vice versa. A negative difference value indicates change in performance under ESK+OAD was worse than change in performance under OAD+PBO and vice versa.

Table 4 shows Pearson correlation coefficients between baseline MADRS total scores and baseline cognitive test Z-scores and between changes in MADRS total scores and changes in cognitive test Z-scores from baseline at Day 28 and the study endpoint in the short-term studies (DB1+DB2 pooled and DB4). At baseline, correlations between depressive symptoms and performance on the cognitive tests were uniformly low (r ≤ 0.3) although CIs for estimates of strength of association did not include zero for the tests of psychomotor function and attention in the younger patients with TRD and for the test of working memory in the older patients. Similarly, in younger patients, correlations between change in depressive symptoms and change in cognition were also uniformly small (r <0.2), and estimates of strength of association whose CIs did not include zero showed no systematic relationship with the ESK treatment. In the older patients, the same pattern of results occurred with most correlations between change in depressive symptoms and change in cognition uniformly small (r <0.2). Although correlations between change in depressive symptoms and change in performance on the ONB and HVLT-R total recall test were moderate in magnitude, with CIs for these estimates not including zero, again these correlations were not specific to the ESK groups.

**Table 4.** Pearson correlation coefficients and 95% CLs for the short-term studies (DB1+DB2 pooled and DB4).

Test	Treatment group	Baseline Z-score			Change from baseline at Day 28			Change from baseline at endpoint		
		N	r	95% CL	N	r	95% CL	N	r	95% CL
DB1+DB2 pooled										
DET	ESK+OAD	332	-0.14	(-0.24, -0.03)	277	-0.10	(-0.22, 0.02)	304	-0.11	(-0.22, 0.01)
	OAD+PBO	212	-0.21	(-0.33, -0.08)	191	-0.07	(-0.21, 0.07)	201	-0.07	(-0.21, 0.07)
IDN	ESK+OAD	337	-0.06	(-0.17, 0.05)	285	-0.19	(-0.3, -0.07)	312	-0.17	(-0.28, -0.06)
	OAD+PBO	214	-0.20	(-0.33, -0.07)	193	-0.19	(-0.32, -0.05)	203	-0.20	(-0.33, -0.06)
OCL	ESK+OAD	339	0	(-0.1, 0.11)	292	-0.02	(-0.14, 0.09)	319	-0.06	(-0.17, 0.05)
	OAD+PBO	218	-0.12	(-0.25, 0.01)	196	0.02	(-0.12, 0.16)	207	0.03	(-0.10, 0.17)
ONB	ESK+OAD	338	-0.02	(-0.13, 0.09)	290	-0.10	(-0.21, 0.02)	317	-0.08	(-0.19, 0.03)
	OAD+PBO	217	-0.14	(-0.26, 0)	195	-0.15	(-0.29, -0.01)	206	-0.16	(-0.29, -0.02)
GML	ESK+OAD	325	-0.04	(-0.15, 0.07)	276	-0.05	(-0.17, 0.07)	302	-0.05	(-0.16, 0.06)
	OAD+PBO	205	-0.08	(-0.22, 0.06)	185	-0.09	(-0.23, 0.06)	195	-0.08	(-0.22, 0.06)
HVLT-R Total Recall	ESK+OAD	343	-0.05	(-0.16, 0.05)	302	-0.16	(-0.26, -0.04)	328	-0.18	(-0.28, -0.07)
	OAD+PBO	221	-0.04	(-0.17, 0.1)	203	-0.03	(-0.17, 0.11)	214	-0.02	(-0.16, 0.11)
HVLT-R_D	ESK+OAD	341	-0.02	(-0.13, 0.09)	300	-0.12	(-0.23, -0.01)	326	-0.15	(-0.25, -0.04)
	OAD+PBO	221	-0.06	(-0.19, 0.07)	203	-0.14	(-0.27, 0)	214	-0.12	(-0.25, 0.02)
DB4										
DET	ESK+OAD	67	-0.27	(-0.48, -0.03)	56	-0.08	(-0.34, 0.18)	61	-0.06	(-0.31, 0.19)
	OAD+PBO	64	-0.25	(-0.47, -0.01)	58	-0.02	(-0.28, 0.24)	60	-0.02	(-0.27, 0.24)
IDN	ESK+OAD	68	-0.30	(-0.5, -0.06)	56	-0.08	(-0.34, 0.19)	61	-0.06	(-0.30, 0.2)
	OAD+PBO	65	-0.15	(-0.38, 0.09)	58	-0.05	(-0.3, 0.21)	60	-0.06	(-0.31, 0.2)
OCL	ESK+OAD	67	-0.16	(-0.39, 0.08)	56	0	(-0.27, 0.26)	61	0	(-0.25, 0.26)
	OAD+PBO	65	-0.09	(-0.33, 0.16)	58	0.22	(-0.04, 0.45)	60	0.19	(-0.06, 0.43)
ONB	ESK+OAD	67	-0.31	(-0.51, -0.08)	58	-0.01	(-0.27, 0.25)	63	0	(-0.25, 0.25)
	OAD+PBO	64	-0.01	(-0.26, 0.23)	56	-0.37	(-0.57, -0.12)	58	-0.27	(-0.49, -0.01)
GML	ESK+OAD	47	0.08	(-0.22, 0.36)	41	0.04	(-0.27, 0.34)	44	0.05	(-0.25, 0.34)
	OAD+PBO	45	0.08	(-0.22, 0.36)	41	0.10	(-0.21, 0.4)	42	0.05	(-0.26, 0.35)
HVLT-R Total Recall	ESK+OAD	71	-0.14	(-0.36, 0.1)	59	-0.30	(-0.51, -0.04)	65	-0.28	(-0.49, -0.04)
	OAD+PBO	65	-0.02	(-0.26, 0)	59	-0.08	(-0.33, 0.18)	61	-0.13	(-0.37, 0.13)
HVLT-R_D	ESK+OAD	71	-0.08	(-0.31, 0.15)	59	-0.05	(-0.3, 0.21)	65	-0.05	(-0.29, 0.19)
	OAD+PBO	65	-0.01	(-0.25, 0.24)	59	-0.13	(-0.37, 0.13)	61	-0.14	(-0.38, 0.12)

Abbreviations: CL, confidence limit; DET, Detection; ESK, esketamine nasal spray; GML, Groton Maze Learning; HVLT-R, Hopkins Verbal Learning Test-Revised; HVLT-R_D, HVLT-R Delayed Recall; IDN, Identification; LOCF, last observation carried forward; OAD, oral antidepressant; OCL, One-Card Learning; ONB, One Back Memory; PBO, placebo nasal spray. Between baseline MADRS total scores and baseline cognitive test Z-scores, and between changes of MADRS total scores and changes of cognitive test Z-scores from baseline at Day 28 and the study endpoint.

N = Number of patients with nonmissing MADRS total scores, baseline cognitive test Z-scores, changes of MADRS total scores and cognitive test Z-scores from baseline at Day 28 and the endpoint. r = Pearson correlation coefficient (range for r is −1 to 1, where r = 0, no correlation; r = (>0) to 1, positive correlation; r = (<0) to −1, negative correlation). Day 28 assessment is the end of the 4-week double-blind phase and endpoint assessments are at LOCF in the double-blind phase.

## DISCUSSION

This analysis of cognitive data, aggregated from the 4 well-controlled phase 3 clinical studies that supported the regulatory approval of ESK, confirmed that short-term or long-term maintenance treatment with ESK was not associated with worsening of psychomotor function, attention, working memory, visual learning, verbal learning and memory, or executive function in patients with TRD. In the short-term (4-week double-blind treatment) studies in younger to mid-life patients (aged 18–64 years) with TRD, a small improvement from baseline was observed for most aspects of cognition in both ESK+OAD and OAD+PBO groups at Day 28 and the study endpoint. Inspection of the signs and magnitudes of the between-treatment group differences in the mean change from baseline Z-scores of cognitive tests (i.e., effect sizes) identified no evidence of a systematic negative effect on any aspect of cognition from short-term treatment with ESK. In fact, for psychomotor function, attention, and memory, small and reliable (CIs did not include zero) treatment-related benefits were observed following short-term ESK+OAD treatment versus OAD+PBO treatment (Table 2). In the long-term maintenance study (DB3), continuation into the double-blind maintenance phase was contingent on patients’ symptomatic response after 16 weeks of the initial treatment (Daly et al., 2019). For patients with TRD treated with ESK who entered the randomized withdrawal phase and received double-blind treatment (i.e., continued ESK or switched to PBO, while continuing OAD), improvement from baseline occurred for each aspect of cognition assessed, except for the attention (IDN for the OAD+PBO group) for both ESK+OAD and OAD+PBO groups at Week 32, 44, 56, and for the study endpoint (Table 3). Again, effect sizes reflecting differences between the ESK and PBO groups also indicated reliable and small-to-moderate magnitude treatment-related benefits for the ESK+OAD group over the OAD+PBO group for attention, working memory, visual learning, and verbal learning and memory (Figure 2; Table 2). While some of the between-treatment group numerical differences on mean change from baseline Z-scores of cognitive tests were “negative” (i.e., favoring the OAD+PBO group), for each of these the 95% CI included zero, indicating that the difference between the ESK+OAD and OAD+PBO groups was not of a magnitude to be considered meaningful.

A further longitudinal analysis of the DB3 study that accounted for actual time in study for each cognitive assessment (including “end of phase” visits) indicated that, despite the declining sample sizes for these visits, the direction and magnitude of treatment effects at Week 32, 44, and 56 (supplementary Table 2) were qualitatively similar to that observed from the main analysis of DB3. The characteristics of the effect of ESK on cognition based on the endpoint analysis did not appear to be influenced by the length of time patients remained in the study. The breadth, consistency, and magnitude of the treatment effects observed in the short- and long-term treatment studies suggests that in patients with TRD, ESK was associated with a small-to-moderate benefit in attention, learning, and memory, with these benefits persisting for patients who experienced a continued antidepressant benefit with ongoing ESK treatment.

For older patients with TRD (aged 65 years or older), improvements from baseline were also observed for working memory and executive function in both ESK+OAD and OAD+PBO groups at Day 28 and study endpoint assessments. However, effect sizes reflecting differences in the change from baseline between the ESK+OAD and OAD+PBO groups, and their associated CIs, indicated there were no significant differences between groups for any aspect of cognition assessed in the older subsample (Table 2; Figure 2). Although the sample size for older patients was smaller compared with younger patients, the consistency of the effect of ESK on cognition, both within and between the two age groups, suggested that the cognitive safety of short-term ESK treatment extended to older patients with TRD.

When considering the large samples studied, the extensive cognitive assessments applied, and the experimental rigor with which the studies were conducted, the current results have considerable clinical importance in confirming that treatment with ESK, either short term or longer term, has no detrimental effects on cognition in patients with TRD. Further, the findings of small-to-moderate benefits in cognition observed in the younger patients suggests, first, that it is possible to improve the cognitive impairment that occurs in TRD and, second, that modulation of NMDA receptors may provide an opportunity to facilitate cognition, in particular, attention and memory processes in patients with TRD.

The large and well-characterized sample of patients with TRD who participated in the Janssen esketamine phase 3 development program and the analytic strategy of expressing performance on each cognitive test in terms of normative data standardized scores also allows understanding of the nature and magnitude of cognitive impairment in TRD. Performance data from the cognitive tests at the baseline visit provide reliable estimates of the magnitude of the cognitive impairment in individuals with TRD experiencing acute illness before beginning study medication. In the younger TRD group (18–64 years), baseline cognitive data indicated the presence of mild-to-moderate magnitude impairment across psychomotor function, attention, working memory, and episodic memory, with these functions being more impaired than visual learning and executive function (Table 2). In the older TRD group (65 years or older), subtle cognitive impairment, qualitatively and quantitatively similar to that in the younger TRD group (group mean standardized scores at baseline in Table 2), was also observed. The subtle cognitive impairment in older adults with TRD was most likely to reflect the effects of the depressive illness in that presence of clinically important cognitive impairment was an exclusion criterion for their entry into DB4. Together, these data suggest that the magnitude of cognitive impairment does not worsen with increasing age or disease chronicity in individuals with TRD. Moreover, the magnitudes of cognitive impairment observed in the current samples were consistent with those observed in other MDD samples, including those with recurrent MDD (Liu et al., 2021). Thus, the baseline data from the esketamine program indicate that while moderate cognitive impairments can be considered an important symptom of TRD, their nature and magnitude suggest they are not distinct from the similar cognitive symptoms in the broader MDD population.

In addition to their mild-to-moderate cognitive impairment, patients in the ESK development program had high levels of depressive symptom severity at enrollment. Despite this, associations between the levels of depressive symptoms and the degree of cognitive impairment were relatively weak in both the younger adult and older TRD groups (Tables 2 and 4), consistent with prior reports (Shilyansky et al., 2016). While short-term treatment with ESK reduced depressive symptoms in TRD (Fedgchin et al., 2019; Popova et al., 2019; Ochs-Ross et al., 2020), the absence of substantial change in cognition over the same period, as well as the absence of any strong and consistent associations between the change in depressive symptoms and the change in cognition related to ESK treatment, suggests some independence between these two treatment effects (Rock et al., 2014; Semkovska et al., 2019; Kriesche et al., 2023) (Table 4) while also raising the possibility that at least some of the ESK-related cognitive benefit may be an indirect consequence of the amelioration of depressive symptoms. While further studies are needed to better evaluate relationships between treatment-related changes in depressive symptoms and cognition, the current findings provide no evidence to suggest that either short-term or long-term treatment with ESK is associated with deterioration in any aspect of cognition.

Of note, change from baseline in performance on the cognitive battery was compared between adults with TRD randomized to treatment with ESK+OAD and OAD+PBO. Thus, any systematic effects of the study design, including those from the repeated application of the neuropsychological tests (i.e., practice effects), will have occurred for individuals receiving ESK and for those receiving PBO. In this context, the impact of practice effects on change from baseline scores on the cognitive tests are controlled. Some limitations in these analyses of cognition data, drawn from the 4 ESK clinical trials, warrant consideration regarding the generalizability of the results. First, in the maintenance phase of the long-term ESK study, patients with TRD had variable treatment durations based on whether they experienced relapse, discontinued treatment early, or the study stopped after the required number of relapses occurred. Therefore, the timing of the study endpoint was not the same for all the patients, limiting the conclusions about the effect on cognition over specific longer-term time intervals. Second, conclusions arising from these analyses were not based on formal inferential analyses of inferiority. As stated, in the ESK development program, cognitive function was treated as a safety endpoint in the phase 3 studies. Hence, no inferential statistics were calculated for submission to the regulatory authorities. To remain consistent with the intent of this study design element, we did not conduct formal tests of statistical significance here. However, the estimates of group raw means and the standardized group mean change from baseline (i.e., Z-scores), and the sample sizes studied, were provided. Estimates of effect sizes to reflect the magnitude of between-group differences as well as 95% CIs for these were computed to guide the interpretation of ESK treatment effects. These data can be used to inform future studies and analyses designed to measure the cognitive safety of new medications for TRD, as well as studies designed to examine relationships between depressive symptoms, cognition, and NMDA receptor antagonism. Also, it should be noted that, though DB3 was a long-term maintenance study of variable duration, the length of exposure may appear relatively short within the context of the typical natural history of depressive disorders, which tend to be lifelong relapsing or remitting conditions for most patients. However, we believe the aggregated results reported here provide clinicians and researchers with a detailed summary of the available information about the effects on cognition of treatment with esketamine in patients with TRD.

In conclusion, these analyses of data aggregated from the 4 controlled ESK phase 3 clinical studies provide a rigorous basis for understanding the extent to which treatment with ESK at therapeutic doses in TRD was associated with effects on cognition. The absence of any negative effects of ESK on cognition informs this aspect of safety of ESK treatment. The findings from evaluable patients with cognitive data (up to 56 Weeks) in the long-term study supported the conclusion that maintenance treatment with ESK also did not result in negative effects on cognition. Consistent with other studies in MDD, cognitive impairments of TRD were related only weakly to levels of depressive symptoms (Shilyansky et al., 2016). In TRD, treatment with esketamine is associated with clinically meaningful reduction in depressive symptoms. While the absence of any cognitive decline arising from treatment with esketamine is also important for patients with TRD, the small benefits to attention and memory observed indicate that there is need for further refinement and development of therapies for treatment of the cognitive impairment that characterizes TRD.

## Supplementary Material

pyae046_suppl_Supplementary_Tables_S1-S2

## Data Availability

The data sharing policy of Janssen Pharmaceutical Companies of Johnson & Johnson is available at https://www.janssen.com/clinical-trials/transparency. As noted on this site, requests for access to the study data can be submitted through Yale Open Data Access (YODA) Project site at http://yoda.yale.edu.

## Interest Statement

Maggie Fedgchin, Keith Karcher, Rosanne Lane, Kim Cooper, and Wayne C. Drevets are employees of Janssen Research & Development, LLC, and may hold company stock and/or stock options. Randall L. Morrison, Jaskaran Singh, Ella Daly, and Rachel Ochs-Ross worked on the clinical development program of esketamine for treatment-resistant depression during their employment by Janssen Research & Development, LLC; Dr Morrison has retired and is currently employed as a consultant to Janssen Research and Development, LLC; Dr Singh is currently employed by Neurocrine Biosciences, San Diego, CA; Dr Daly is currently a self-employed medical consultant; Dr Ochs-Ross is currently self-employed. Paul Maruff is an employee of Cogstate Ltd, Melbourne, Australia. Gerard Sanacora has served as consultant to Abbvie, Ancora/Embark, Aptinyx, Atai, Axsome Therapeutics, Biogen, Biohaven Pharmaceuticals, Boehringer Ingelheim International GmbH, Bristol-Myers Squibb, Clexio, Cowen, Denovo Biopharma, Daiichi Sankyo, ECR1, EMA Wellness, Clexio, Denovo Biopharma, ECR1, EMA Wellness, Freedom Biosciences, Gilgamesh, Intra-Cellular Therapies, Janssen, KOA Health, Levo therapeutics, Lundbeck, Merck, MiCure, Navitor Pharmaceuticals, Neurocrine, Novartis, Noven Pharmaceuticals, Perception Neuroscience, Praxis Therapeutics, Relmada Therapeutics, Sage Pharmaceuticals, Seelos Pharmaceuticals, Taisho Pharmaceuticals, Usona Institute, Valeant, Vistagen Therapeutics, and XW Labs; and received research contracts from Johnson & Johnson/Janssen, Merck, and the Usona Institute over the past 36 months. Dr Sanacora holds equity in Biohaven Pharmaceuticals, Freedom Biosciences, Gilead, Relmada, and Tetricus. In addition, he is a co-inventor on a US patent (#8,778,979) held by Yale University and a co-inventor on US Provisional Patent Application No. 047162-7177P1 (00754) filed on August 20, 2018, by Yale University Office of Cooperative Research. Yale University has a financial relationship with Janssen Pharmaceuticals and may receive financial benefits from this relationship. The University has put multiple measures in place to mitigate this institutional conflict of interest. Questions about the details of these measures should be directed to Yale University’s Conflict of Interest office.
