# Supplementary material for: Effect of Esketamine Nasal Spray on Cognition in Patients With Treatment-Resistant Depression: Results From Four Phase 3 Studies
Source: Int J Neuropsychopharmacol. 2024 Nov 8;27(11):pyae046. doi: 10.1093/ijnp/pyae046 (PMC11561565; doi:10.1093/ijnp/pyae046)
Supplement: pyae046_suppl_Supplementary_Tables_S1-S2 [file pyae046_suppl_supplementary_tables_s1-s2.docx]

1. Longitudinal analysis of study DB3 maintenance phase

In the maintenance phase endpoint analysis of study DB3, the timing of the study endpoint was not the same for all patients. Cognitive assessments at early withdrawal visits (e.g., due to relapse) frequently occurred prior to the first scheduled maintenance phase cognitive assessment visit at Week 32. As an additional analysis of the maintenance phase of Study DB3, assessment visits, including early withdrawal visits, were assigned to time points based on visit windows defined by the relative day of the visit in the maintenance (MA) phase and between-group comparisons were made with a longitudinal analysis utilizing these time points.

Visit windows

MA phase visits, including early withdrawal visits, were windowed based on relative day in the MA phase. Visit windows were labelled assuming the MA phase began at study Week 16.

If multiple visits were assigned to the same time point, the visit closest to the target day for that timepoint was used in the analysis.

Baseline was the same study baseline used for the endpoint analysis.

Supplementary Table 1: Analysis visit windows

| **Time point** | **Relative day^a^** | **Target day** |
| --- | --- | --- |
| Week 20 (MA) | 2-49 | 29 |
| Week 26 (MA) | 50-91 | 71 |
| Week 32 (MA) | 92-155 | 113 |
| Week 44 (MA) | 156-239 | 197 |
| Week 56 (MA) | 240-323 | 281 |
| Week 68 (MA) | 324-407 | 365 |
| Week 80 (MA) | 408-491 | 449 |
| Week 92 (MA) | ≥492 | 533 |
| ^a^Relative to start of maintenance phase. | | |

Between-group comparisons by MMRM

Change from baseline for each cognition test was analyzed using a mixed effects model for repeated measures (MMRM) with change from baseline as the dependent variable, factors for maintenance phase treatment group, country, time point (as defined above), and treatment group-by-time point interaction; and baseline z-score of the test as a covariate. A first-order autoregressive [AR(1)] variance-covariance structure was used; the model did not converge with unstructured or Toeplitz covariance structures. The Kenward-Roger method was used for approximating the denominator degrees of freedom. Least-squares means and 95% confidence intervals of the between-group differences at each time point were estimated from the MMRM.

Results

Descriptive statistics for cognitive test z-scores and changes from baseline at maintenance phase Weeks 32, 44, and 56, as defined above, are summarized in Supplementary Table 2 below. The sample at each time point includes patients with assessments at those scheduled visits and any early withdrawal visits that fell in the window defined for that visit. Descriptive statistics for baseline at each time point is based on the baseline values for those patients with observations at the given time point. Between-group differences and 95% confidence intervals estimated from the MMRM are also provided.

Supplementary Table 2: Cognitive Test Z-scores: Change from Baseline at Maintenance Phase Weeks 32, 44, and 56 of Study DB3

|  |  |  | **Baseline Assessment** | | | **Visit Assessment** | | | **Change from Baseline** | | | **ESK+OAD minus OAD+PBO difference^a^** | | |
| --- | --- | --- | --- | --- | --- | --- | --- | --- | --- | --- | --- | --- | --- | --- |
| **Test** | **Time point** | **Group** | **N** | **Mean** | **SD** | **N** | **Mean** | **SD** | **N** | **Mean** | **SD** | **Estimate** | **Lower 95% CL** | **Upper**  **95% CL** |
| DET | Week 32 (MA) | ESK+OAD | 79 | -1.27 | 1.913 | 79 | -0.73 | 1.511 | 79 | 0.54 | 1.545 | 0.14 | -0.26 | 0.54 |
|  |  | OAD+PBO | 54 | -1.17 | 1.756 | 54 | -0.73 | 1.258 | 54 | 0.44 | 1.588 |  |  |  |
|  | Week 44 (MA) | ESK+OAD | 51 | -1.6 | 2.062 | 51 | -0.65 | 1.169 | 51 | 0.95 | 1.85 | 0.48 | 0.01 | 0.95 |
|  |  | OAD+PBO | 36 | -1.25 | 1.882 | 36 | -0.94 | 1.563 | 36 | 0.31 | 1.045 |  |  |  |
|  | Week 56 (MA) | ESK+OAD | 30 | -1.29 | 2.328 | 30 | -0.64 | 1.379 | 30 | 0.65 | 1.9 | 0.66 | -0.01 | 1.34 |
|  |  | OAD+PBO | 14 | -1.39 | 2.346 | 14 | -1.35 | 2.12 | 14 | 0.04 | 2.256 |  |  |  |
| IDN | Week 32 (MA) | ESK+OAD | 81 | -1 | 1.928 | 81 | -0.51 | 1.454 | 81 | 0.49 | 1.397 | 0.02 | -0.38 | 0.43 |
|  |  | OAD+PBO | 55 | -0.92 | 1.815 | 55 | -0.46 | 1.526 | 55 | 0.46 | 1.63 |  |  |  |
|  | Week 44 (MA) | ESK+OAD | 53 | -1.28 | 2.015 | 53 | -0.76 | 1.398 | 53 | 0.52 | 1.513 | 0.13 | -0.34 | 0.6 |
|  |  | OAD+PBO | 37 | -1 | 1.871 | 37 | -0.83 | 1.787 | 37 | 0.17 | 1.064 |  |  |  |
|  | Week 56 (MA) | ESK+OAD | 30 | -1.07 | 2.086 | 30 | -0.55 | 1.295 | 30 | 0.52 | 1.451 | 0.77 | 0.08 | 1.45 |
|  |  | OAD+PBO | 14 | -1.13 | 2.195 | 14 | -1.23 | 2.418 | 14 | -0.11 | 1.545 |  |  |  |
| OCL | Week 32 (MA) | ESK+OAD | 84 | -0.66 | 1.353 | 84 | -0.15 | 1.293 | 84 | 0.51 | 1.522 | 0.11 | -0.28 | 0.5 |
|  |  | OAD+PBO | 57 | -0.48 | 1.293 | 57 | -0.2 | 1.222 | 57 | 0.28 | 1.32 |  |  |  |
|  | Week 44 (MA) | ESK+OAD | 55 | -0.82 | 1.319 | 55 | -0.17 | 1.228 | 55 | 0.65 | 1.5 | 0.04 | -0.41 | 0.48 |
|  |  | OAD+PBO | 37 | -0.66 | 1.266 | 37 | -0.16 | 1.304 | 37 | 0.5 | 1.263 |  |  |  |
|  | Week 56 (MA) | ESK+OAD | 30 | -0.6 | 1.075 | 30 | -0.22 | 1.126 | 30 | 0.39 | 1.184 | -0.16 | -0.8 | 0.48 |
|  |  | OAD+PBO | 14 | 0 | 0.887 | 14 | 0.46 | 1.105 | 14 | 0.47 | 0.926 |  |  |  |
| ONB | Week 32 (MA) | ESK+OAD | 83 | -0.91 | 1.597 | 83 | -0.45 | 1.139 | 83 | 0.46 | 1.223 | 0.4 | 0.06 | 0.73 |
|  |  | OAD+PBO | 57 | -0.8 | 1.463 | 57 | -0.83 | 1.371 | 57 | -0.04 | 1.089 |  |  |  |
|  | Week 44 (MA) | ESK+OAD | 54 | -1.1 | 1.71 | 54 | -0.43 | 1.149 | 54 | 0.67 | 1.34 | 0.28 | -0.12 | 0.68 |
|  |  | OAD+PBO | 37 | -1.03 | 1.623 | 37 | -0.76 | 1.568 | 37 | 0.27 | 0.962 |  |  |  |
|  | Week 56 (MA) | ESK+OAD | 30 | -0.97 | 1.742 | 30 | -0.26 | 0.904 | 30 | 0.72 | 1.314 | 0.71 | 0.13 | 1.3 |
|  |  | OAD+PBO | 14 | -0.91 | 1.637 | 14 | -0.97 | 2.333 | 14 | -0.05 | 1.616 |  |  |  |
| GML | Week 32 (MA) | ESK+OAD | 79 | -0.09 | 0.991 | 79 | 0.2 | 1.162 | 79 | 0.29 | 1.184 | -0.13 | -0.44 | 0.19 |
|  |  | OAD+PBO | 53 | -0.33 | 1.791 | 53 | 0.23 | 0.803 | 53 | 0.56 | 1.345 |  |  |  |
|  | Week 44 (MA) | ESK+OAD | 49 | -0.09 | 1.028 | 49 | 0.26 | 0.839 | 49 | 0.35 | 0.867 | -0.25 | -0.61 | 0.11 |
|  |  | OAD+PBO | 34 | -0.24 | 0.73 | 34 | 0.26 | 0.775 | 34 | 0.5 | 0.696 |  |  |  |
|  | Week 56 (MA) | ESK+OAD | 27 | -0.06 | 1.03 | 27 | 0.32 | 0.899 | 27 | 0.37 | 1.065 | 0.17 | -0.34 | 0.67 |
|  |  | OAD+PBO | 13 | -0.15 | 0.645 | 13 | 0.35 | 0.74 | 13 | 0.5 | 0.801 |  |  |  |
| HVLT-R Total Recall | Week 32 (MA) | ESK+OAD | 89 | -1 | 1.298 | 89 | -0.17 | 1.23 | 89 | 0.83 | 1.019 | 0.42 | 0.09 | 0.75 |
|  |  | OAD+PBO | 60 | -1.1 | 1.438 | 60 | -0.67 | 1.365 | 60 | 0.43 | 1.112 |  |  |  |
|  | Week 44 (MA) | ESK+OAD | 59 | -0.93 | 1.303 | 59 | -0.1 | 1.274 | 59 | 0.83 | 1.111 | 0.3 | -0.09 | 0.68 |
|  |  | OAD+PBO | 37 | -1.2 | 1.373 | 37 | -0.49 | 1.49 | 37 | 0.71 | 1.059 |  |  |  |
|  | Week 56 (MA) | ESK+OAD | 29 | -0.92 | 1.224 | 29 | 0.11 | 1.216 | 29 | 1.03 | 0.996 | 0.53 | -0.03 | 1.09 |
|  |  | OAD+PBO | 14 | -1.26 | 1.39 | 14 | -0.6 | 1.476 | 14 | 0.67 | 0.944 |  |  |  |
| HVLT-R_D | Week 32 (MA) | ESK+OAD | 89 | -1.04 | 1.281 | 89 | -0.25 | 1.115 | 89 | 0.79 | 1.091 | 0.44 | 0.12 | 0.76 |
|  |  | OAD+PBO | 60 | -1.06 | 1.284 | 60 | -0.71 | 1.346 | 60 | 0.35 | 1.138 |  |  |  |
|  | Week 44 (MA) | ESK+OAD | 59 | -1.05 | 1.362 | 59 | -0.25 | 1.22 | 59 | 0.8 | 1.088 | 0.15 | -0.23 | 0.52 |
|  |  | OAD+PBO | 37 | -1.1 | 1.307 | 37 | -0.35 | 1.333 | 37 | 0.75 | 0.838 |  |  |  |
|  | Week 56 (MA) | ESK+OAD | 29 | -1.09 | 1.434 | 29 | -0.07 | 1.136 | 29 | 1.01 | 1.057 | 0.66 | 0.11 | 1.21 |
|  |  | OAD+PBO | 14 | -0.76 | 2.797 | 14 | -0.63 | 1.371 | 14 | 0.13 | 2.33 |  |  |  |
| ^a^Based on MMRM with maintenance phase treatment group, country, week, and treatment group-by-week interaction as factors and baseline z-score as a covariate.  CL, confidence limit; DET, Detection; ESK, esketamine nasal spray; GML, Groton Maze Learning; HVLT-R, Hopkins Verbal Learning Test-Revised; HVLT-R_D, HVLT-R Delayed Recall; IDN, Identification; OAD, oral antidepressant; OCL, One-Card Learning; ONB, One Back Memory; PBO, placebo nasal spray; SD, standard deviation; | | | | | | | | | | | | | | |
